# Personalized Nutrition in Pediatric Chronic Diseases

**DOI:** 10.3390/metabo15100653

**Published:** 2025-09-30

**Authors:** Marlene Escobedo-Monge, Robert H. Lustig, Sergey Suchkov, Sofia Blokh, Natalya Andronova, Olga Goryacheva, Marina Borisovna Moyseyak, Timur Vlasov, Arturo Solís Herrera, Veronika Polyakova, Elena Antonova, Aleksandr Tuykavin

**Affiliations:** 1 Department of Pediatrics, Faculty of Medicine, University of Valladolid, Avenida Ramón y Cajal, 7, 47005 Valladolid, Spain; 2 International Cooperation for Health and Social Development (CIDESS) Group, University of Las Palmas de Gran Canaria, Juan de Quesada Street, 30, 35001 Canary Islands, Spain; 3 Valladolid Health Research Institute (IBioVALL), C. Rondilla Sta. Teresa, 47010 Valladolid, Spain; 4 Division of Endocrinology, Department of Pediatrics, University of California San Francisco, San Francisco, CA 94143, USA; robert.lustig@ucsf.edu; 5 Center for Biodesign, N.D. Zelinskii Institute for Organic Chemistry of the Russian Academy of Sciences, Moscow 119991, Russia; 6 China Hong Kong Innovation International Business Association, Hong Kong, China; 7 The New York Academy of Sciences, New York, NY 10006, USA; 8 The European Association for Predictive, Preventive and Personalized Medicine (EPMA), 1160 Brussels, Belgium; 9 International Society of Personalized Medicine, Tokyo 102-0073, Japan; 10 Personalized Medicine Coalition (PMC), Washington, DC 20036, USA; 11 American Chemical Society (ACS), Washington, DC 20036, USA; 12 General Hospital Department, N.F. Filatov’s Moscow Clinical Pediatric Hospital, Moscow 123001, Russia; child82@list.ru; 13 Department Moscow City, Grand Clinic Moscow, Moscow 123112, Russia; andronowa@mail.ru; 14 Department of Propaedeutics of Childhood Diseases, Pediatric Faculty, Institute of Maternal and Childhood, N.I. Pirogov Russian National Research Medical University, Moscow 117997, Russia; doc.equimos@gmail.com; 15 Gastroenterology Department, Morozovskaya Children’s City Clinical Hospital, Moscow 119049, Russia; 16 Institute of Food Systems and Health-Saving Technologies, Faculty of Technology of Fermentation and Winemaking, Moscow 109004, Russia; marina-mgupp@mail.ru; 17 Department of Pathophysiology with Course in Clinical Pathophysiology, Faculty of Medicine Pavlov First Saint Petersburg State Medical University, Saint Petersburg 197022, Russia; tvlasov@yandex.ru (T.V.); atuykavin@mail.ru (A.T.); 18 Institute of Human Photosynthesis, Aguascalientes 20000, Mexico; comagua2000@gmail.com; 19 Department of Personalized Medicine, Precision Nutrition and Biodesign, Institute of Biotechnology and Global Health, BIOTECH University, Moscow 125080, Russia; med_nika2000@mail.ru; 20 Department of Statistics, N.F. Filatov’s Moscow Clinical Pediatric Hospital, Moscow 123001, Russia; lenikantonova@gmail.com

**Keywords:** personalized and precision medicine, multi-omics, microbiota

## Abstract

This narrative review examines the application of personalized nutrition (PN) through multi-OMICS and trans-OMICS in pediatric populations, particularly in relation to chronic conditions such as obesity, type 2 diabetes, and celiac disease. We synthesize evidence to identify biomarkers and gene–environment interactions and translate molecular insights into individualized dietary guidance. Even though PN represents a promising strategy for optimizing child health, significant challenges remain in translating molecular findings into practical, cost-effective, and equitable interventions. We advocate integrating this knowledge into clinical practice and developing policies and standardized methodologies that ensure accessibility for all pediatric populations.

## 1. Introduction

The global rise in chronic diseases underscores the profound impact of unhealthy diets and sedentary lifestyles. Alarmingly, these conditions are increasingly diagnosed in childhood, with obesity as a harbinger of multiple noncommunicable diseases (NCDs). Obesity itself represents an independent risk factor for type 2 diabetes (T2DM), insulin resistance (IR), dyslipidemia, and cancer [1]. In the United States, the proportion of children aged 5–17 years with chronic conditions or functional limitations increased from 22.6% in 1999–2000 to 30.2% in 2017–2018—equivalent to approximately 130,000 additional children affected each year [2]. These findings emphasize the urgent need for innovative nutritional strategies beyond conventional population-based guidelines.

**Figure 1:**
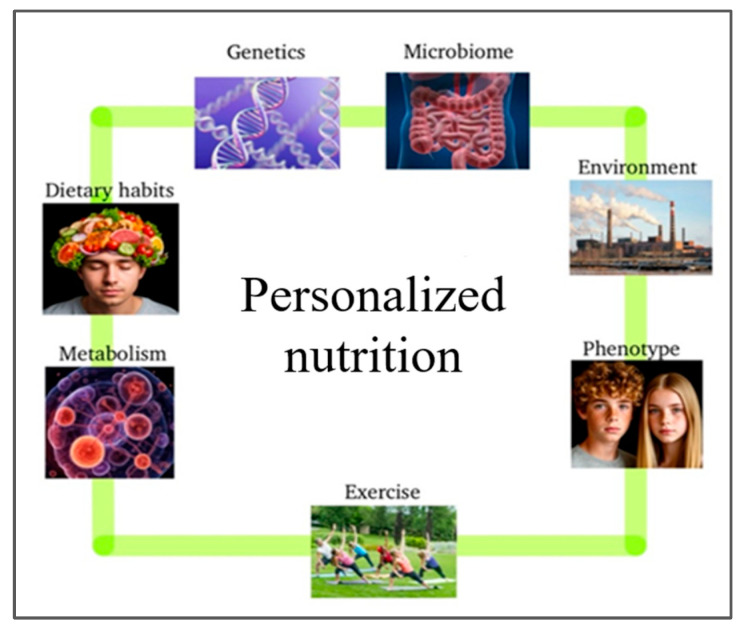
Personalized nutrition uses individual data and OMICS technologies to tailor diets, revealing how genetic, phenotypic, and environmental factors can influence nutrient absorption and action, and ultimately disease risk [7,8].

Personalized nutrition (PN) offers a promising approach by tailoring dietary interventions with individual biological, lifestyle, and socio-demographic profiles [3,4]. Unlike traditional nutrition, PN integrates diverse factors—including socioeconomic status, ethnicity, health history, genetics, and gender—to deliver targeted recommendations [5,6]. Advances in OMICS technologies now enable more precise analyses of gene–diet interactions, gut microbiome compositions, and metabolic responses, revealing inter-individual variability in nutrient processing [3]. For example, genotype-specific variants can significantly alter nutrient metabolism; thus, dietary guidance can be modified. Figure 1 shows the specific data involved in developing such PN algorithms.

This paradigm shift from generalized to individualized recommendations is particularly relevant in pediatrics. Nutritional needs vary across developmental stages, and early-life exposure—including prenatal factors—profoundly modifies long-term health trajectories. Given the rising burden of pediatric chronic diseases, adapting dietary guidance for children and adolescents represents both a challenge and an opportunity for prevention and disease management. Therefore, this review examines the role of PN in pediatric obesity, T2DM, and celiac disease (CD), with a focus on how individualized approaches can contribute to improved prevention, treatment, and long-term health outcomes.

## 2. Methods

We conducted a comprehensive literature search of publications from June 2024 to August 2025, using major electronic databases, including PubMed, Google Scholar, ScienceDirect, Web of Science, and the Cochrane Library. Studies were eligible for inclusion if they focused on individuals 19 years of age or under, including pregnant adolescents. Exclusion criteria included individuals over 19 and lactating women.

A combined search was performed, using the same Boolean logic in all engines. The dates were restricted to 1 January 2020 through 31 May 2025: (“personalized nutrition” OR “precision nutrition” OR “nutrition-guided approaches”) AND (obesity OR “diabetes mellitus” OR T1DM OR T2DM OR “type 1 diabetes” OR “type 2 diabetes” OR “celiac disease”) AND (infants OR children OR adolescents OR “pregnant adolescents” OR “pediatric populations” OR paediatr*) AND (OMICS OR omics OR metagenomic* OR metabolom* OR proteom* OR nutrigenom* OR microbiota OR “gut microbiome”). We considered only studies published in English or with English-language abstracts.

We identified a total of 878 records through database searches: PubMed (n = 32), Clarivate (n = 32), Web of Science (n = 39), Cochrane Library (n = 1), and Google Scholar (n = 774). Among the records from Google Scholar, 177 were reviews. After removing duplicates, we screened the titles and abstracts for relevance. We excluded studies that included the search terms but did not address the primary research aim from the analysis. The remaining articles made up the majority of the references included in this review. We added additional information from authoritative sources such as the World Health Organization (WHO) and the United Nations Children’s Fund (UNICEF). We did not conduct a formal risk-of-bias assessment.

## 3. Personalized Nutrition in Pediatric Populations

### 3.1. The Human Microbiome in Pediatric Chronic Conditions

In recent decades, the prevalence of chronic diseases—including respiratory, allergy, autoimmune, metabolic, and psychiatric disorders—has increased markedly, especially in industrialized countries. This trend is closely linked to urbanization, lifestyle changes, and increasing exposure to environmental stressors, such as reduced biodiversity, chemical agents, polyaromatic hydrocarbons, microplastics, and endocrine disruptors, which can destabilize the immune and endocrine systems, in part by altering the host and environmental microbiomes [9,10]. The rapidity of these changes has exceeded the adaptive capacity of the human immune system, fomenting new mechanisms involved in chronic disease pathogenesis, such as intestinal dysbiosis, chronic immune dysregulation, and mild systemic inflammation [11].

Despite a growing body of evidence, causal pathways between microbial alterations and disease remain poorly defined. While in vivo and in vitro studies link gut microbiome variation to conditions such as obesity, rheumatoid arthritis, T2DM, Alzheimer’s disease, Parkinson’s disease, and depression, robust mechanistic insights and clinical translation remain limited. Notably, fecal microbiota transplantation for recurrent *Clostridioides difficile* infection stands as a rare example of microbiome-targeted intervention with proven efficacy [12]. This situation underscores both the therapeutic potential and the current limitations of microbiome-based strategies. Table 1 summarizes key studies investigating the role of gut microbiome in pediatric diseases.

Pediatric microbiome research focuses on the interplay between immune, metabolic, and neurological development during early childhood—a critical window of microbial plasticity and modifiability. Unlike in adults, the infant microbiome is less influenced by accumulated external confounders, making prospective postnatal studies particularly valuable for exploring causal relationships and guiding long-term preventive strategies in immunity, metabolism, and neurodevelopment [22]. Large-scale cohorts such as TEDDY (The Environmental Determinants of Diabetes in the Young), DIABIMMUNE, and COPSAC (Copenhagen Prospective Studies on Asthma in Childhood) have advanced this field; however, each has inherent methodological strengths and limitations, as summarized in Table 2.

Can these findings inform clinical practice? The COPSAC study found that early bacterial colonization of the neonatal airways by *S. pneumoniae*, *H. influenzae*, or *M. catarrhalis* is associated with transient early onset wheezing and asthma, emphasizing the need to minimize early exposure to respiratory pathogens [18]. The Northern European study revealed that Finnish and Estonian infants, who have higher rates of autoimmune diseases, possess more *Bacteroides* species and genes related to lipopolysaccharide (LPS) biosynthesis, producing a form of LPS that dampens immune activation—suggesting impaired immune education compared to control Russian infants [14,15]. Meanwhile, the TEDDY study shows that as infants transition from breastfeeding to solid foods, their gut microbiome matures from milk-fermenting to fiber-metabolizing activity, and that factors like age, geography, and antibiotic use significantly influence microbial stability [13]. These results highlight the essential role of microbial exposure during early life and demonstrate how factors such as nutrition, environmental conditions, and clinical interventions can influence it.

Table 3 summarizes dietary interventions proposed in the TEDDY study to reduce the risk of T1DM, including exclusive breastfeeding for six months, cautious timing of solid foods, limiting early gluten and cow’s milk exposure, prudent antibiotic use with possible probiotic support, and high-fiber diets to boost SCFA production. Beyond these specific measures, broader preventive approaches highlight the importance of microbiome-focused nutrition, judicious antibiotic stewardship, and the identification of microbial and metabolic markers for early risk prediction. Critically, integrating exposomic and genomic data holds promise for developing individualized strategies to enhance immune resilience from early life.

**Figure 2:**
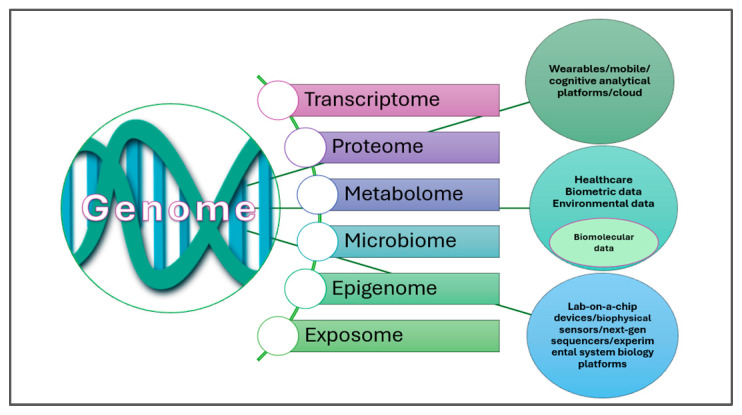
Personalized and precision medicine integrates genomic, exposomic, and clinical data. While wearable devices enable real-time ecological and biometric monitoring, capturing high-resolution biomolecular data remains a major challenge for systems biology research [24].

Many NCDs share underlying risk factors such as gut dysbiosis, chronic immune dysfunction, and low-grade inflammation. Immune-mediated diseases (IMDs), including allergies, asthma, T1DM, and CD, are of particular concern due to their rising prevalence, chronic course, and impact on quality of life and life expectancy. IMDs arise from a breakdown of immune tolerance to self or harmless antigens, leading to inflammation and tissue damage. Current therapies are mainly symptomatic, costly, and often associated with adverse effects, with allergen desensitization being one of the few partially curative options [23]. Figure 2 illustrates how Personalized and Precision Medicine (PPM) contextualizes data, ranging from genomic information to exposomic factors.

PPM contextualizes genomic and exposomic data to characterize disease risk and heterogeneity. The exposome encompasses general external factors (e.g., socioeconomic conditions, climate), specific external exposures (e.g., pathogens, pollutants, lifestyle), and internal factors (e.g., microbiota, immune activity, metabolism, hormones) [11]. Critically, early-life exposures within these domains can predispose to or protect against IMDs, depending on gene–environment and epigenetic interactions [25]. Emerging OMICS technologies and molecular diagnostics offer promise for identifying mechanistic pathways and enabling tailored prevention or treatment [26,27]. Epigenetic modifications—shaped by early-life microbiota through mechanisms such as DNA methylation and histone modification—play a central role in immune maturation and IMD susceptibility. Since genetic predisposition alone cannot explain the increasing incidence of IMDs, greater emphasis must be placed on environmental and exposomic drivers [28,29].

### 3.2. Personalized Nutrition via the Resources of Precision Foodomics

**Figure 3:**
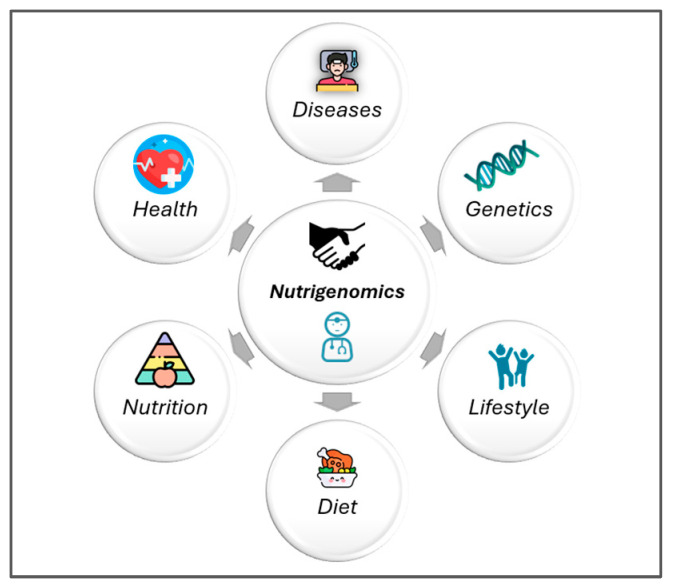
Nutrigenomics and nutrigenetics examine how diet interacts with the genome, influencing gene expression and individual metabolic responses, providing a scientific basis for personalized nutrition strategies tailored to genetic profiles [32].

PN uses metabolomics and nutrigenomics to tailor diets to individual needs, offering more precise chronic disease prevention than generalized guidelines by elucidating how diet influences disease onset and progression (Figure 3) [30,31].

Even though PN shows considerable potential, its application in pediatric populations remains limited, with most research focusing on adults [33,34]. Adult studies, such as the PREVENTOMICS project, demonstrate that metabolomic, proteomic, and genetic dietary recommendations can positively influence diet choices, even when metabolic markers remain unchanged [34]. These findings highlight the potential of PN to influence behavior and suggest its value in identifying individuals who may be more receptive to specific dietary interventions. In children, PN has begun to show benefits, including reductions in malnutrition and improvements in health outcomes, as seen in interventions targeting early childhood nutrition, cerebral palsy, and obesity through individualized dietary, behavioral, and physical strategies, such as the Etiology-based Personalized Intervention Strategy for Childhood Obesity (EPISTCO) approach [35,36,37].

**Figure 4:**
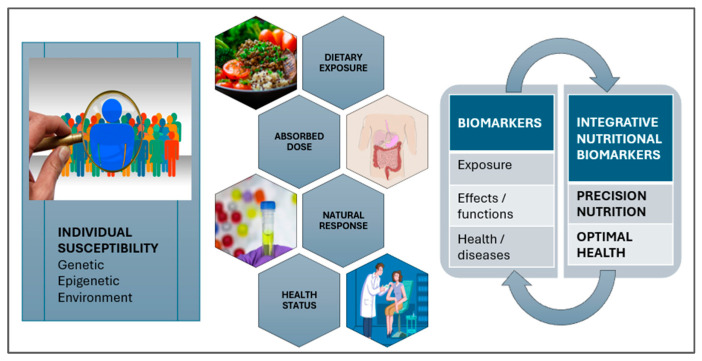
Integrative nutritional biomarkers, revealed through OMICS technologies, link diet to biological responses and health outcomes, enabling more precise and individualized nutrition strategies [43,44].

Multi-omics integration—combining microbiome, metabolomics, and genomics—offers powerful tools to refine these interventions, though most algorithmic platforms remain unapproved for routine pediatric use. Commercial tests (e.g., Nutrigenomix) provide Single Nucleotide Polymorphisms (SNP)-based dietary recommendations [38,39], while validated biomarkers (Figure 4) can assess fiber fermentation and obesity risk early in life [40]. While TEDDY predicts the risk of autoimmune diseases in children with T1DM, COPSAC utilizes metabolomic and microbiome data to guide early-life nutrition for asthma and allergy, and LifeLines DEEP/PREDICT (The Personalized REsponses to DIetary Composition Trial) extensions investigate adolescent postprandial responses based on gut profiles [41,42]. Collectively, these developments illustrate a critical shift toward individualized, data-driven pediatric nutrition, while highlighting the need for further clinical validation and standardized implementation.

**Figure 5:**
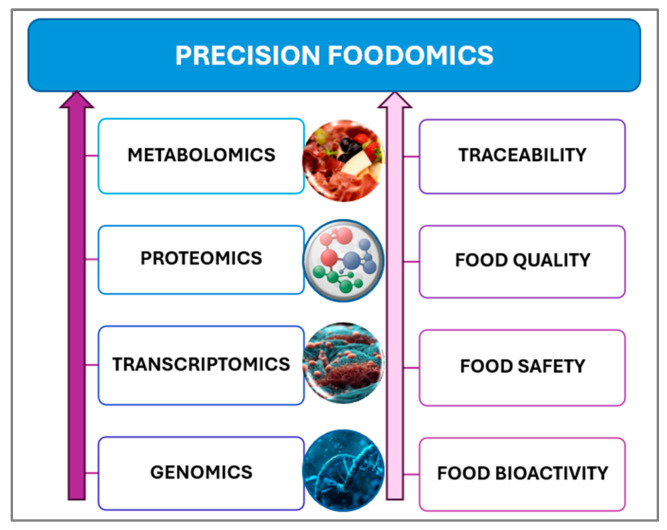
Precision Foodomics (PF) uses OMICS technologies to link food composition with individual molecular responses, supporting personalized nutrition strategies tailored to unique biological profiles [46].

Advances in OMICS technologies have propelled the emergence of Precision Foodomics (PF) (Figure 5) and data-driven food design, making PN increasingly feasible [45]. By integrating genomics, microbiomics, and metabolomics, PN—through nutrigenetics—can tailor dietary interventions and supplements to individual biological profiles [32]. PF enables the identification of objective biomarkers for food intake and health status, supporting real-time monitoring and personalized recommendations [44]. Leveraging bioinformatics and IT tools, PF assesses gene–diet–metabolite interactions, particularly the interplay between the gut microbiome, metabolome, and diet, to guide the development of functional foods. Understanding food composition and chemical diversity remains essential for evidence-based, sustainable nutrition, as PF links dietary components directly to health and disease outcomes [46].

Advances in genomics, including candidate gene approaches and genome-wide association studies (GWAS), have identified numerous SNPs influencing nutrient digestion, metabolism, and signaling pathways [47,48]. GWAS has also explored complex behavioral traits such as fussy eating in children, which can progress to avoidant/restrictive food intake disorder (ARFID) with nutritional, medical, and psychological consequences. Although a study across three European cohorts (4391 cases, 16,460 controls) suggested potential genetic links to autism and anorexia nervosa, the associations were not statistically significant [49], highlighting the need for larger, more targeted investigations.

**Figure 6:**
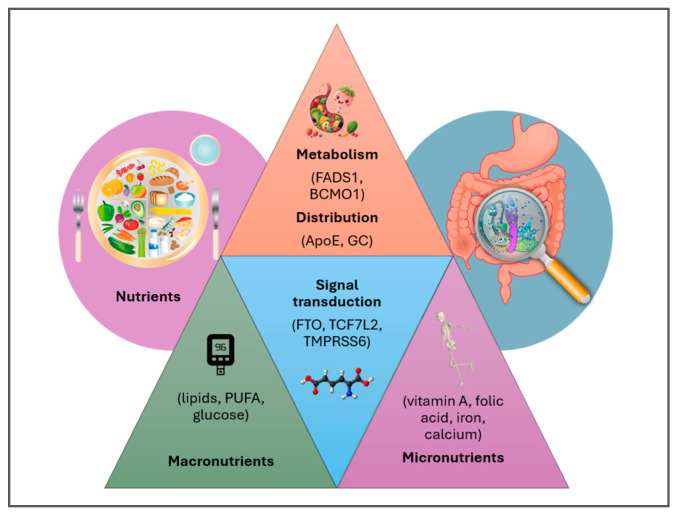
High-throughput technologies in Personalized and Precision Medicine reveal nutrient–gene interactions, enabling individualized diagnostics, treatments, and preventive strategies to reduce disease burden [50].

Within this context, genotype-informed dietary recommendations—integrating key clinical parameters—offer targeted support for individuals with metabolic or nutritional vulnerabilities, enhancing the precision of PN (Figure 6) [50]. Epigenetic studies on maternal diet and infant DNA methylation have shown inconsistent results due to small sample sizes, methodological heterogeneity, and limitations in dietary assessment tools (e.g., FFQs, biomarkers) [51,52]. These findings underscore the gap between OMICS-based research and practical pediatric guidance.

**Figure 7:**
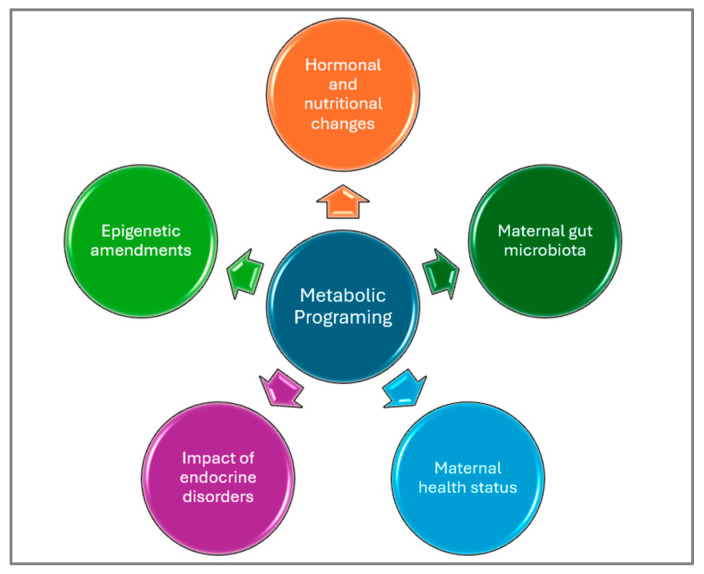
Metabolic programming links early-life nutrition, epigenetic changes, and microbial interactions to long-term health, influencing susceptibility to metabolic disorders in adulthood [53].

On the other hand, metabolic programming and metabolic imprinting define early life events, with impacts on later physiological outcomes (Figure 7).

The development of chronic diseases is due to hereditary, epigenetic, and reversible mechanisms that affect genetic expression without altering DNA sequences. Nutrition during early life, including maternal and postnatal diets, has long-term effects via epigenetic programming. This influences metabolic pathways and disease susceptibility; a mechanism referred to as metabolic programming or imprinting [31]. While targeted nutritional interventions during critical developmental windows are promising, current genomics-based PN faces limitations in accounting for interactions among genetic predisposition, dietary behaviors, microbiota composition, and environmental exposures [54]. Addressing these multifactorial complexities requires standardized, longitudinal, and integrative studies to implement tailored nutrition advice.

## 4. Personalized Nutrition in Chronic Diseases

Below, we review recent advances in PN and PF for pediatric healthcare, settling on complex conditions such as autoimmunity and hormonal imbalances, and other pediatric health challenges. While these data-driven, individualized strategies hold promise, their clinical translation is limited by gaps in evidence, standardization, and ethical guidance, highlighting the need for cautious, well-designed implementation in children.

### 4.1. Obesity

Pediatric obesity is a complex, multifactorial condition shaped by both nutritional deficiencies and broader genetic, environmental, and socioeconomic determinants [55]. While macro- and micronutrient imbalances contribute to obesity and the early onset of chronic diseases [56,57,58,59,60,61], current treatment approaches remain limited—focusing mainly on lifestyle modifications, with minimal pharmacological innovation compared to adult care [62]. It is important to note that the increasing rates of childhood obesity cannot be explained solely by genetics. Environmental and socioeconomic disparities, particularly among low-income populations, play a pivotal role by limiting access to healthy food and physical activity [55]. Recent multi-omics research (e.g., the Human Early Life Exposome project) underscores the biological complexity of obesity, revealing inflammation-driven metabolic dysfunction linked to both pre-pregnancy maternal health and environmental toxins [63]. Despite growing evidence, public health efforts often fail to address the systemic and early-life drivers of obesity, limiting the effectiveness of interventions for at-risk populations.

Assessing energy requirements in pediatric populations—especially among obese or critically ill children—remains a significant clinical challenge due to the limitations of traditional predictive methods such as resting energy expenditure (REE) equations and indirect calorimetry, which are often imprecise or impractical [33,64]. Recent advances in artificial intelligence (AI) and machine learning (ML), including Artificial Neural Networks (ANNs), metabolomics, and microbiota analysis, offer more personalized and potentially accurate approaches for estimating energy needs and guiding interventions [33,65]. These AI-powered tools are increasingly used to support diagnosis, treatment planning, and prevention in pediatric care, including through interactive platforms such as gamified nutrition apps and social robot systems [66]. Studies by Triantafyllidis and Larizza have demonstrated high engagement [67,68]. However, many of these models, despite their potential, are still in the initial stages and require broader, long-term validation for use in clinical practice.

While AI offers exciting possibilities for individualized pediatric nutrition care, we must also acknowledge several limitations. Many models rely on narrow datasets and oversimplified evaluation metrics such as area under the receiver operating characteristic curve (AUROC) or accuracy, which may not adequately reflect real-world performance [69]. A shift toward more comprehensive evaluation strategies, including metrics like the F1 score and MetaMetrics, is necessary to improve model reliability and applicability [70]. Though promising, microbiota-based predictive tools (e.g., AUROC: 0.73) have yet to prove clinical utility. Data heterogeneity in pediatric populations, lack of standardization, and challenges in integrating complex, real-world information further hinder implementation [66]. Moreover, ethical, legal, and social issues—particularly surrounding genomics—pose significant barriers, including concerns over informed consent, data privacy, and long-term implications of predictive testing in children [71,72]. Addressing these challenges requires robust, ethically sound frameworks, standardized data practices, and inclusive approaches that incorporate behavioral and environmental factors.

In this sense, technology-delivered PN interventions have shown promising outcomes in adult populations by improving dietary habits, such as reducing energy and fat intake while increasing vegetable consumption. These interventions utilize a range of digital platforms (apps, wearables, SMS, social networks, etc.) to deliver personalized recommendations based on behavioral, genetic, and phenotypic data [73]. However, despite the apparent benefits, the overall quality of evidence remains low, limiting confidence in their efficacy and generalizability [73,74]. The extensive heterogeneity in intervention designs, coupled with methodological limitations in the underlying studies, weakens the case for immediate clinical adoption. It is essential for the pediatric population to design future interventions more rigorously and validate them thoroughly to ensure consistent and replicable results.

In pediatric populations, the challenge of adherence remains a principal barrier to success, often undermining the potential benefits of nutritional or lifestyle interventions. The one-size-fits-all model overlooks interindividual variability, especially in a developmental context, where motivation, discipline, and environmental factors (e.g., family support, school environment) play critical roles. While tools like mobile apps and gamified platforms may boost engagement, their impact on long-term behavior change is uncertain without sustained support and personalization [37]. Thus, while technology-enhanced PN has potential, particularly for improving access and personalizing care, its success in pediatrics depends on overcoming adherence barriers and adapting strategies to developmental, social, and behavioral realities.

On the other hand, AI in PN remains underdeveloped, with a primary focus on dietary assessment rather than broader applications such as malnutrition prediction, behavior change, or disease prevention [75]. While lifestyle and diet interventions, particularly those aligned with the Mediterranean diet, show theoretical potential for addressing pediatric obesity, practical results are inconsistent. For example, despite improvements in diet and physical activity using tools such as the BigO platform, changes in BMI were negligible, highlighting a disconnect between behavior modification and measurable clinical outcomes [76,77]. This issue raises questions about the sensitivity of short-term interventions and the need for more robust long-term evaluation frameworks.

Additionally, evidence for technology-assisted interventions in children shows modest improvements when combined with traditional care, especially after at least six months of follow-up [78]. However, methodological limitations and small sample sizes continue to restrict the generalizability of these findings. While the role of epigenetics suggests that early environmental influences can have lasting biological impacts, translating this knowledge into potent and scalable pediatric interventions is still a challenge [52]. Current lifestyle-based strategies are inadequate in many cases, and while pharmacotherapy shows potential, its safety and efficacy in children remain poorly established [37]. Overall, the promise of AI, big data, and epigenetic knowledge in pediatric obesity remains limited by a lack of scientific evidence, a deficient clinical translation, and limited long-term outcomes.

### 4.2. Type 2 Diabetes Mellitus

From a critical perspective, pediatric T2DM is a rapidly growing public health challenge, primarily motivated by rising childhood obesity and lifestyle factors. Despite the known severity of its long-term sequelae—such as early-onset renal failure, amputations, and vision loss—data on pediatric prognosis remains scarce, limiting the ability to predict or mitigate outcomes fully [79]. The fact that pediatric T2DM patients face a disproportionately higher risk of dialysis (40-fold) compared to their peers underscores both the aggressiveness of the disease and the urgency for earlier, more targeted interventions. Furthermore, current management heavily relies on extrapolating data from adults, yet pediatric T2DM may follow a distinct and more aggressive clinical course, making this approach inadequate [80]. The lack of robust longitudinal studies, combined with variability in adherence to treatment and healthy lifestyle changes, hampers progress in reducing complications. Without systematic screening, comprehensive patient and family education, and integrated multidisciplinary intervention, pediatric T2DM is responsible for early morbidity and mortality, which healthcare systems will be ill-equipped to address.

Traditional glycemic markers, such as fasting glucose and hemoglobin A_1C_ (HbA_1C_), have diagnostic and prognostic limitations in T2DM, as they do not detect transient hyperglycemia, and factors like comorbidities and ethnicity influence their results [81]. Alternative markers—glycated albumin, fructosamine, and 1,5-anhydroglucitol—offer independent, clinically valuable data and outperform conventional indices in predicting the risk of T2DM, as demonstrated in the Atherosclerosis Risk in Communities (ARIC) study [82]. In addition, advances in glucose monitoring, including the Glucose Monitoring Indicator (GMI), continuous glucose monitoring (CGM), and closed-loop systems [automated insulin delivery (AID) systems], allow for more precise and automated insulin adjustments and improved treatment customization [81]. Integration with mobile devices further enhances self-care, real-time feedback, and data sharing, marking a shift toward more dynamic and individualized DM management.

Non-conventional biomarkers better capture postprandial fluctuations and the effects of oxidative stress that HbA_1C_ does not. This issue highlights the need for a multi-timescale approach to glycemic monitoring—short, medium, and long term—through the combined use of diverse biomarkers to improve prevention, early detection, and management of diabetes [83]. However, such integration remains underutilized in clinical practice, limiting the precision and timeliness of interventions. In pediatric T2DM prevention, recommendations emphasize early-life strategies such as exclusive breastfeeding for the first 4–6 months and family-based dietary habits from age one, prioritizing plant-based foods while minimizing high-sugar and high-salt products [84]. Despite strong epidemiological links between diet patterns, obesity, and later glucose intolerance, genetic–nutrient interaction studies remain inconclusive at scale, with only a few variants (e.g., TCF7L2, GIPR, CAV2, PEPD) showing possible associations with specific macronutrient responses [30]. This gap highlights a persistent challenge: without integrating genetic insights, environmental exposures, and precise biomarker monitoring, current prevention strategies risk staying broad and insufficiently personalized.

Pediatric-onset T2DM poses a much greater clinical challenge than its adult counterpart, as obese children face a fourfold higher risk and a markedly faster decline in β-cell function, insulin sensitivity (despite excessive insulin secretion), and a more rapid progression of microvascular and cardiovascular complications than youth with T1DM or adults with T2DM. Despite international guidelines emphasizing lifestyle interventions such as PN, daily physical activity for at least 60 min, and behavioral modification, clinical practice outcomes remain poor, as evidenced by the high treatment failure rates of the Treatment Options for Type 2 Diabetes in Adolescents and Young Adults (TODAY) trial, which reported high failure rates for metformin alone (52%), combination therapy (38.6%), and lifestyle modification (46.6%) [85]. This situation highlights a critical deficiency in current strategies. While theoretically sound, they fall short of the aggressiveness for early-onset disease. Without early detection, more targeted treatment algorithms, and interventions that go beyond generic lifestyle recommendations, treatment of pediatric T2DM risks being reactive rather than truly preventative.

**Figure 8:**
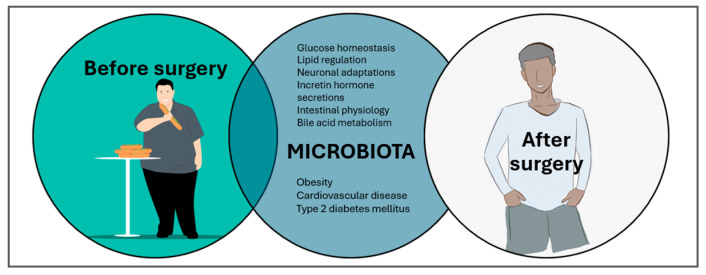
Vertical sleeve gastrectomy (VSG) and Roux-en-Y gastric bypass (RYGB) alter gut microbiota, but the mechanisms linking these changes to metabolic benefits remain unclear, limiting the development of microbiota-targeted therapies [89,90].

Bariatric surgery (Figure 8), while increasingly recommended for obese adults with T2DM due to its marked improvements in glycemic control, lipid homeostasis, incretin secretion, bile acid modulation, and gut–brain signaling, remains a controversial intervention in pediatrics [86]. Even though its ability to promote intestinal and neuronal adaptations and alter gut microbiota is well established, the long-term safety for pediatric patients and the psychosocial implications are still insufficiently explored. Emerging evidence links gut microbial dysbiosis to increased serum zonulin—a marker of intestinal permeability—elevated HOMA-IR, and higher endotoxemia markers (e.g., LPS-binding protein) [87]. In overweight and obese adolescents, zonulin correlates with IR, although findings remain inconsistent across studies, underscoring the complexity of gut metabolism interactions [88]. The developing pediatric gut microbiota is vulnerable to dietary or surgical disruptions, potentially affecting obesity-related comorbidities through mechanisms not yet fully understood.

**Figure 9:**
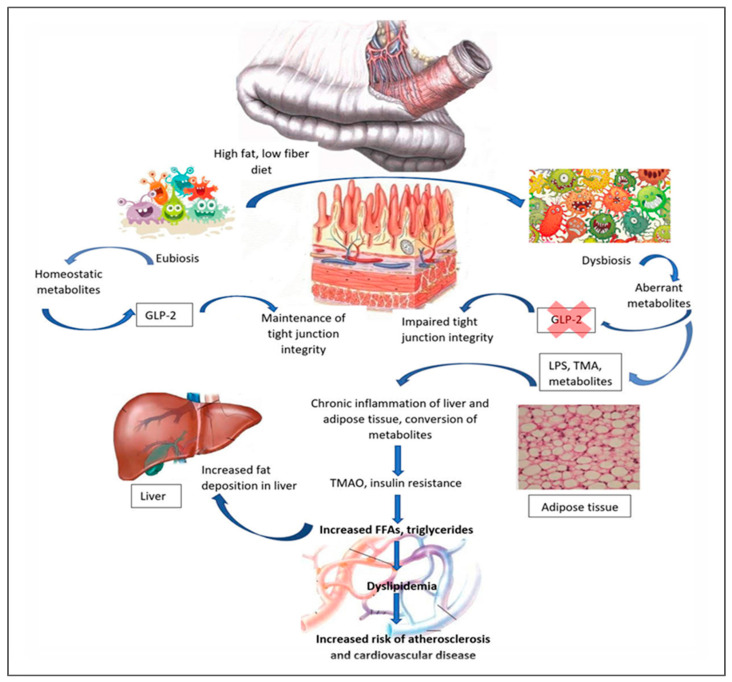
The intestinal microbiome closely interacts with the regenerative epithelial barrier, shaping metabolic disease risk through influences on immune responses, barrier integrity, and systemic communication. Microbial composition, diet, hormones, inflammation, and neural signals collectively modulate this interface, while metabolites produced near the epithelium—such as FFAs, GLP-2, LPS, TMA, and TMAO—play key roles in keeping gut and overall health [94,95,96]. Abbreviations: FFA: Free fatty acid; GLP-2: Glucagon-like peptide-2; LPS: Lipopolysaccharide; TMA: Trimethylamine; TMAO: trimethylamine N-oxide.

Dietary quality plays a critical role in this axis. Low-fiber diets reduce microbial-derived SCFAs, impairing anorectic hormone release, anti-inflammatory cytokine production, and mucin synthesis—key components of intestinal barrier integrity [91]. SCFAs, via GPR41 and GPR43 signaling, not only regulate appetite but also enhance intestinal gluconeogenesis, influencing systemic glucose homeostasis. Conversely, high-fat diets promote metabolic endotoxemia via LPS translocation, activating TLR4-mediated inflammatory cascades that drive IR and pathogenesis of T2DM [92]. Thus, while bariatric surgery offers rapid metabolic benefits, its role should be contextualized within a broader strategy that addresses pediatric-specific gut–immune–metabolic dynamics, prioritizing microbiota-supportive dietary interventions to complement or potentially reduce the need for invasive approaches. Establishing causality between gut microbiota adaptations and childhood obesity is essential but remains challenging [93] (Figure 9).

The gut microbiome has emerged as a modifiable factor in the pathogenesis of T2DM, to improve insulin sensitivity and restore gut barrier function [97]. Mendelian randomization analyses from large European GWAS–microbiome cohorts found 16 genera, including *Bacteroides* and *Clostridium*, as potentially causal. However, we may not generalize these findings to other ethnicities [98]. While mouse studies link obesity to *Firmicutes*-rich, *Bacteroidetes*-poor microbiota, human evidence is less definitive [99]. While diet–microbiota interactions, particularly fiber-driven SCFA production that enhances GLP-1/GLP-2 signaling, hold promises in protecting gut integrity and metabolic health, high-fat, low-fiber diets promote microbial metabolite-driven inflammation [100]. However, current data remain primarily associative, limiting translation into targeted microbiome-based therapies.

In the human intestinal microbiota, Firmicutes (60–80%) and Bacteroidetes (20–30%) predominate, playing a protective role against obesity, metabolic syndrome, and T2DM. Obesity often results in reduced microbial diversity, which promotes IR, inflammation, and fat accumulation [101]. Associations between specific taxa—such as *Prevotella copri* and *Bacteroides vulgatus*—and IR have been linked to increased production of branched-chain amino acids (BCAAs), tryptophan metabolites, and LPS, all implicated in metabolic disease [91]. However, findings on Bacteroides abundance in obesity remain inconsistent: obese adults often show higher Bacteroides and lower Firmicutes [102], while obese children display the opposite pattern, reversible with dietary interventions [103]. Microbiome profiling may predict how individuals respond to diets; for example, those who are Prevotella-dominant tend to respond better to high-fiber diets. However, this approach remains experimental, costly, and lacks strong causal trial data. In T2DM, reduced butyrate-producing bacteria and elevated BCAA-producing species are common [104]. Modifying the Prevotella/Bacteroides ratio through targeted diets (e.g., barley) can improve postprandial glucose, supporting a personalized microbiome-based approach [105].

### 4.3. Type 1 Diabetes

Type 1 diabetes mellitus is a systemic autoimmune disease characterized by the destruction of pancreatic β-cells, resulting in inadequate insulin production and impairing glucose, lipid, protein, and mineral metabolism. Although commonly presented in childhood, the extent of β-cell loss varies, with some individuals keeping minimal insulin secretion after onset [106]. Its pathogenesis is strongly influenced by genetic predisposition, although proposed environmental triggers—such as viral infections, cow’s milk proteins, and vitamin D3 deficiency—remain associative rather than causally proven, limiting prevention strategies. Diagnosis relies on autoimmune biomarkers, including anti-islet cell, anti-glutamate decarboxylase (GAD), anti-insulin, anti-tyrosine phosphatase, and anti-ZnT8 antibodies. However, we do not know the mechanistic link between immune activation and environmental factors [107]. This incomplete understanding, combined with disease heterogeneity, highlights the need for more precise characterization to enable targeted and personalized interventions.

Changes in intestinal tight junction proteins—such as claudin-2, occluding, cingulin, and ZO proteins—combined with elevated zonulin levels, can increase gut permeability and appear before the clinical onset of T1DM, suggesting a possible early biomarker of disease risk [108]. These permeability shifts, modulated by bacterial colonization, intensify the hypothesis that gut microbiota modifications contribute to T1DM pathogenesis [109]. Multi-omics and multicenter studies have revealed microbial patterns associated with the disease, notably a decreased Firmicutes/Bacteroidetes ratio and an overrepresentation of Bifidobacterium, the latter associated with a higher risk of T1DM [110]. While such findings advance understanding and hold translational potential, causality remains unproven, and the clinical application of microbiome-based prognostic tools remains limited by methodological variability, pediatric population bias, and the need for longitudinal validation.

Metagenomic–metabolomic analyses in T1DM reveal a consistent microbial and metabolic imbalance. The enrichment of *Clostridiales* and *Dorea*, depletion of *Dialister* and *Akkermansia*, and elevated metabolites like isobutyrate and malonate. Notably, high GAD antibody titers correlate with reduced *Roseburia*, *Faecalibacterium*, and *Alistipes*—key butyrate producers—while preserved metabolic control (normal HbA_1C_) aligns with elevated purine/pyrimidine intermediates [107]. The reduction in butyrate-producing species (*Clostridium* groups IV/XIVa) and mucin-degrading species (*Prevotella*, *Akkermansia*) is associated with increased intestinal permeability and a higher risk of T1DM, which negatively affects the protective metabolic and barrier functions of SCFAs [111,112]. Although sodium butyrate supplementation shows promise in improving IR, the mechanistic basis—particularly the molecular triggers linking microbial shifts, permeability changes, and β-cell autoimmunity—is still poorly defined, limiting translation into predictive or therapeutic tools. 

Nutrition in early life, including prolonged breastfeeding and late introduction of solid foods, is critical for shaping a healthy gut microbiome and may reduce the risk of T1DM [113]. Premature dietary shifts can induce dysbiosis, impair immune maturation, and predispose to β-cell autoimmunity. While probiotics, prebiotics, and postbiotics—particularly *Lactobacillus*, *Bifidobacterium*, and *Akkermansia muciniphila*—show promise in enhancing gut barrier integrity, reducing inflammation, and modulating metabolism, robust preventive evidence is limited due to individual variability in diet, genetics, and environment [101]. Decreased SCFA-producing bacteria, such as *Roseburia* and *Faecalibacterium*, correlate with early T1DM markers, highlighting the therapeutic potential of the microbiome modulation [114]. Current T1DM management remains centered on insulin, though adjunctive strategies—including closed-loop systems, SGLT2 inhibitors, immune therapies, and β-cell replacement—are under investigation [115]. Despite metabolic and anti-inflammatory benefits observed with probiotics in trials, their integration into standard care requires stronger mechanistic evidence and long-term outcome validation.

T1DM often coexists with other autoimmune disorders, including thyroid disorders, parathyroid disorders, CD, vitiligo, gastritis, dermatological conditions, and rheumatic diseases [116]. Notably, CD affects 3–16% of patients (mean 8%). This comorbidity reflects shared genetic risk and overlapping immune dysregulation, with CD often remaining asymptomatic yet detectable via serological markers such as tissue transglutaminase antibodies [117]. Given the potential for silent disease and its impact on prognosis, routine screening at T1DM diagnosis is critical. Management in confirmed cases requires strict adherence to a gluten-free diet (GFD), while asymptomatic, seropositive individuals without histological changes warrant close monitoring to avoid unnecessary dietary restrictions. Critically, failure to find and manage coexisting CD risks compounds systemic inflammation, nutrient malabsorption, and glycemic instability, further complicating T1DM outcomes.

### 4.4. Celiac Disease

Celiac disease is an autoimmune disorder triggered by gluten in genetically predisposed individuals, causing intestinal inflammation and structural damage [118]. While its immune mechanisms are understood, the drivers of intestinal remodeling remain unclear [119]. A strict lifelong GFD is the only treatment [120]. However, adherence is difficult due to social stigma, limited food availability, nutritional gaps, and excessive costs, especially in diverse or resource-limited settings [121]. Despite proven benefits of early, strict GFD adherence, it often results in deficiencies of essential micronutrients, requiring regular nutritional monitoring and supplementation [122]. This issue highlights that while dietary habits early in life can influence disease risk, the highest clinical priority lies in systematic screening, prompt diagnosis, and comprehensive nutritional management to prevent long-term complications.

While CD diagnosis in children relies on highly sensitive and specific serological markers—particularly antibodies against tissue transglutaminase (anti-tTG), endomysium (EMA), and antibodies against deamidated gliadin peptides (anti-DPG)—critical limitations remain [123]. Anti-tTG, despite its 98% sensitivity and specificity, is prone to false positives in other autoimmune, oncologic, hepatic, cardiovascular, and dermatologic conditions, which can compromise diagnostic accuracy [124]. EMA testing offers comparable precision but is resource-intensive, requiring specialized equipment and expertise, limiting its accessibility. Anti-DPG provides only marginal added value and is better reserved for cases with elevated anti-tTG [125]. Rapid point-of-care tests (POC) help prompt detection but may lack the robustness of standard assays. Novel biochemical markers, such as NOx and Tcys, show potential for assessing disease activity, although their clinical utility remains to be validated [126]. Overall, reliance solely on serology without integrating clinical, histological, and genetic data risks overdiagnosis or misclassification, underscoring the need for a multimodal diagnostic strategy.

Emerging evidence highlights the gut microbiota as a critical environmental factor in the pathogenesis of CD, interacting with genetic predisposition and gluten exposure to drive the loss of tolerance and increased intestinal permeability [127]. Dysbiosis—characterized by reduced probiotic taxa (*Bifidobacterium* spp., *Lactobacillus* spp., *Faecalibacterium prausnitzii*) and elevated proinflammatory bacteria (*Bacteroides* spp., *Escherichia coli*, *Staphylococcus* spp.)—appears in both untreated CD and patients on a GFD [128]. Mechanistically, gut microbes can mimic human transglutaminase activity or modulate tight junction proteins and zonulin release, directly influencing barrier function. However, inconsistencies in species-specific findings and the persistence of dysbiosis despite dietary intervention suggest a complex, bidirectional relationship that remains incompletely understood [129]. While small-scale studies suggest microbiome-driven metabolic shifts that could inform targeted therapies, larger and more robust investigations are essential before translating these insights into clinical interventions.

It is crucial to highlight that adherence to a GFD and dietary differences between countries influence the composition of gut microbiota in patients with CD. However, variability in practices makes it difficult to draw definitive conclusions. While a long-term GFD can restore the integrity of the intestinal epithelium and stabilize the microbiome, there are no clear or universal microbial changes, highlighting the need for more robust data [130]. Notably, pediatric patients appear to experience more complete gut recovery compared to adults. However, even after treatment, the number of key probiotic bacteria, such as *Bifidobacterium*, stays at lower levels than in healthy controls, suggesting an incomplete restoration of the microbiota. The increase in *Lactobacillus* in patients on a GFD raises questions about its role and whether dietary interventions fully restore a healthy microbial balance. Furthermore, the uncertain role of probiotics advocates for cautious use given their potential to alter microbiota, but without solid clinical evidence [131]. Overall, the impact of a GFD on the microbiome is promising but far from definitive, highlighting the need for standardized dietary protocols and larger longitudinal studies to clarify how diet shapes microbial recovery in CD.

## 5. New Perspectives

In this section, we present emerging perspectives on PN in some chronic childhood conditions.

### 5.1. Childhood Obesity

The current dominance of purely data-driven strategies (apps, wearables, and digital diaries) will ultimately give way to contextual models that integrate social determinants of health, including family dynamics, food insecurity, sleep quality, and emotional well-being. Such models could enhance predictive accuracy and ensure interventions are relevant to disadvantaged and high-risk children. Recent perspectives on childhood obesity emphasize a shift from one-size-fits-all approaches toward personalized, systemic, and technology-driven solutions. Pharmacotherapy, such as GLP-1 receptor agonists like liraglutide, has shown promise in weight loss and reducing mealtime conflicts when combined with standard intensive lifestyle programs [132], while policy measures, implementing a ban on advertising junk food, aim to decrease risk at the population level; for example, junk food advertising is prohibited in the United Kingdom.

Advances in AI and predictive modeling allow for the early identification of children at risk, easing prompt and targeted interventions. Meanwhile, gamification, IoT, and social robotics platforms are appearing as compelling tools for driving sustained behavior change. Serious games integrated into federated frameworks (combining health apps, AI, biobanks, and gamified education) are proving effective in improving BMI knowledge and outcomes in young people [133]. Complementary approaches use IoT ecosystems (smart devices, wearable sensors, environmental monitoring) to collect real-time behavioral data and support scalable interventions [134]. Together, these strategies underscore a transition toward dynamic, personalized, and ethically grounded ecosystems that integrate healthcare, families, schools, and communities to improve long-term outcomes.

### 5.2. Childhood Type 2 Diabetes Mellitus

New insights into T2DM therapy in children emphasize the importance of initiative-taking, personalized, and multifaceted approaches. Recent advances include the development of AI-based predictive tools, such as Aire-DM, made by the NHS. In adults, this app analyzes routine ECG data to predict T2DM risk up to 13 years before onset, enabling earlier lifestyle interventions [135]. Another innovative direction is the use of digital twin technologies, such as GlyTwin, which simulate individualized behavioral and therapeutic scenarios using counterfactual reasoning to optimize glycemic control, an approach with great potential in adolescents [136]. Similarly, AI-based insulin delivery systems enhance dosing accuracy, although concerns about algorithmic transparency and child-specific safety remain [137]. Finally, school-based prevention initiatives, such as the Kids and Diabetes in Schools (KiDS) program led by the International Diabetes Federation, seek to promote early education, reduce stigma, and encourage healthier lifestyles in children and adolescents [138]. These insights extend beyond BMI and glucose metrics, emphasizing the importance of ahead-of-detection, microbiome-targeted strategies, digital personalization, and community engagement to revolutionize the prevention and management of pediatric T2DM.

### 5.3. Childhood Type 1 Diabetes Mellitus

Emerging perspectives on T1DM are evolving towards a multidimensional framework that includes environmental, immunological, microbial, and technological factors. For example, enteroviruses, particularly *Coxsackie B*, are increasingly implicated as environmental triggers through mechanisms such as molecular mimicry and persistent pancreatic infection [139]. Targeted immunomodulation is advancing, and anti-CD3 monoclonal antibodies, such as teplizumab, have proven delayed disease onset and improved C-peptide preservation, particularly when administered to high-risk individuals before clinical onset [140]. Technological innovation in glycemic control, such as hybrid and closed-loop artificial pancreas systems using AI and deep learning, holds promise for optimizing insulin delivery with greater precision and reducing user burden [141]. Meanwhile, regenerative and β-cell-sparing strategies, including stem cell and encapsulation technologies, are becoming a clinical reality, although long-term efficacy and immunoprotection require further validation [142]. Collectively, these innovations are promising, but many are still in the exploratory phase, with efficacy shown mainly at early stages or in controlled settings, highlighting the pressing need for rigorous translational studies to translate these concepts into evidence-based preventive and therapeutic strategies.

### 5.4. Childhood Celiac Disease

Childhood CD management is evolving from a one-size-fits-all GFD toward precision medicine approaches—integrating early-life interventions, omics-based diagnostics, microbiome modulation, and novel adjunctive therapies, though robust validation in pediatric populations is still needed. Recent research highlights a paradigm shift in childhood CD from strict gluten exclusion alone toward preventive, personalized, and adjunctive strategies. Advances in metabolomics and microbiome profiling show promise for early detection, with distinct signatures detectable years before clinical onset [143]. Prebiotics, probiotics, and synbiotics may enhance gut barrier integrity and reduce inflammation in children on a GFD, though strain-specific effects require further study [144]. Beyond gluten intolerance, CD is increasingly recognized as part of a broader immune dysregulation, with frequent comorbid allergies and immunoglobulin deficiencies, suggesting the need for expanded screening [145]. Finally, emerging therapies such as gluten-degrading enzymes (latiglutenase) and zonulin inhibitors aim to complement dietary management but remain experimental [146].

## 6. Discussion

Compared to adults, PN in pediatric populations with chronic diseases such as obesity, T1DM, T2DM, and CD has received less coverage and has proved a more limited impact. This gap is primarily explained by the scarcity of randomized controlled trials in children, with existing studies being small and of very low certainty, in contrast to the more robust adult literature [147]. The rapid growth, hormonal changes, and developmental variability of children add further complexity, making it hard to identify standardized biomarkers and interventions [33]. Moreover, most validated nutritional biomarkers and omics-based tools have been developed in adults, leaving pediatric applications underexplored [148]. Ethical and practical challenges, such as obtaining consent, respecting child autonomy, and the need for long-term monitoring, also limit clinical translation. Finally, unequal access to PN tools—often resource-intensive and concentrated on high-income settings—risks widening health disparities in children [149]. These limitations underscore the urgent need for large-scale, longitudinal, and pediatric-specific studies to bridge the gap between experimental PN models and clinical practice.

Nutrition is fundamental to pediatrics, yet moving beyond conventional assessments to integrate nutrigenomics, metagenomics, and metabolomics faces barriers of cost, standardization, and limited pediatric validation [150,151]. Although the Barker hypothesis and DOHaD framework suggest early nutrition influences lifelong health, much evidence remains correlational rather than causational [152]. Nutrigenomics holds preventive potential by clarifying gene–nutrient interactions [153], but its translation into pediatric interventions is still nascent. Moreover, evidence from adult PN management cannot be directly applied to children without age-specific data [154,155,156]. Overall, while OMICS-driven approaches in pediatric nutrition are promising, their clinical adoption requires stronger longitudinal evidence and ethical safeguards.

While PN holds great promise in tailoring dietary recommendations to individual genetic, metabolic, and lifestyle characteristics, global accessibility and equity challenges hamper its large-scale impact. Despite its potential, PN remains largely unreachable to low-income populations due to cost, lack of insurance coverage, and limited availability of trained professionals, leaving the majority without access to individualized interventions [157,158]. Meanwhile, global efforts by WHO and UNICEF focus on combating the triple burden of malnutrition—undernutrition, micronutrient deficiencies, and obesity—particularly in vulnerable populations where PN solutions are least available [159]. Although food-based dietary guidelines (FBDG) provide broad, evidence-based public health strategies, PN could refine them by integrating cultural, socioeconomic, and phenotypic diversity, as well as new tools such as nutrigenomics, metagenomics, and biomarker discovery [160,161,162]. However, regulatory frameworks remain fragmented: the EU regulates PN through existing legislation such as Regulation (EC) No 1924/2006 on health and nutrition claims [163,164], while the U.S. lacks specific regulations, creating a market where companies make broad, unverified claims, undermining scientific credibility [165]. Therefore, while PN could revolutionize preventive nutrition, we must address its ethical, regulatory, and equity challenges before it can truly serve as a global public health tool.

**Figure 10:**
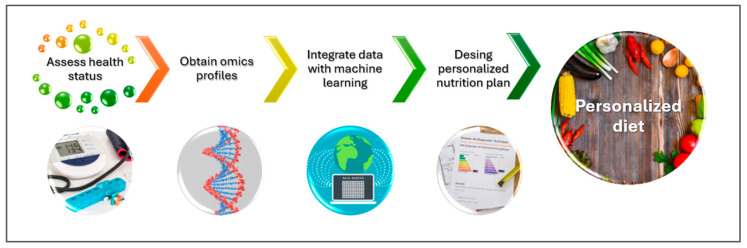
Translate OMICS data into dietary plans in the pediatric population. Personalized pediatric nutrition combines OMICS and clinical data, using machine learning to create tailored dietary and behavioral recommendations that support optimal growth and long-term health.

In pediatric populations, PN offers the potential to optimize growth, development, and long-term health, with promising applications for both healthy children and those at risk for chronic diseases. Integrating PN with PF, functional foods, and systems biology can illuminate host–microbiome interactions, pathogen resistance, immunology, feeding mechanisms, and nutrient utilization, while linking genetic and environmental responses to fundamental biological networks (Figure 10).

In theory, this allows health professionals to deliver child-centered interventions through the following:

- Tailored dietary guidance aligned with individual growth patterns and metabolic profiles.
- Provision of nutrient-adequate meals that support immune function and healthy development.
- Expert oversight from PN specialists to optimize pediatric interventions.

Emerging perspectives in PN emphasize the integration of genomic, metabolomic, and microbiome data with environmental and lifestyle factors to redefine chronic disease prevention and management strategies. In cases of obesity and T2DM, nutrigenomics and metabolomics play key roles in identifying gene–diet interactions that affect metabolic risks and responses to specific dietary interventions. In autoimmune diseases such as CD or T1DM, PN approaches guided by microbiome composition and immune biomarkers enable personalized elimination diets and microbiota-supportive nutrition, improving outcomes beyond standard protocols.

Nonetheless, applying PN in children raises several critical concerns. A strong theoretical foundation is still lacking to decide which traits—genetic, metabolic, or behavioral—are truly relevant for personalization in growing individuals, where nutritional needs vary rapidly across developmental stages. Successful implementation of PN depends on robust frameworks that find relevant individual traits, confirmed integrative biomarkers, and rigorous study designs that demonstrate efficacy, cost-effectiveness, and feasibility in family-centered settings. Regulatory frameworks that protect the privacy and data of children are equally essential to fostering trust and ethical practice. However, challenges persist, such as rapid developmental changes that complicate the identification and interpretation of biomarkers. Socioeconomic disparities can limit access, while inaccurate dietary assessment and inconsistent methods threaten the reliability of interventions.

Potential risks include restrictive dietary practices, reduced dietary diversity, and unintended effects on microbiome development. Ethical considerations related to consent, commercialization, and data privacy highlight the importance of exercising caution. While PN holds promise for pediatric health, its translation into clinical or public health practice requires holistic, ethically grounded strategies that address methodological limitations, potential risks, and equitable access. Moreover, the emphasis on “personalized” strategies should not overshadow broader determinants of child nutrition, such as food insecurity, cultural dietary practices, and the obesogenic environment. Only then can PN fulfill its potential to improve childhood nutrition and long-term well-being. Otherwise, PN risks becoming an elitist tool—scientifically innovative but clinically limited in its ability to address the nutritional needs of children.

## 7. Conclusions

Personalized nutrition, in addition to offering the potential to improve pediatric health by supporting growth, development, and long-term disease prevention, can tailor diets to the genetic, microbiological, and environmental profiles of children with chronic childhood diseases. In obesity and type 2 diabetes mellitus, nutrigenomics and metabolomics provide insights into gene–diet interactions that influence metabolic risk and dietary responsiveness. Similarly, in autoimmune conditions such as celiac disease and type 1 diabetes mellitus, microbiome- and immune-based PN approaches offer opportunities to refine elimination diets and introduce microbiota-supportive interventions that may surpass standard care. Despite these advances, their translation into clinical practice remains limited. Key barriers include the lack of validated pediatric biomarkers, the challenges of adapting interventions during periods of rapid growth, and structural inequities that restrict access and risk, exacerbating health disparities. Ethical issues—ranging from informed consent and child autonomy to data protection—further complicate widespread adoption. For PN to achieve meaningful impact in pediatrics, it must transition from individualized, resource-intensive models toward accessible, evidence-based frameworks that are family- and community-oriented, underpinned by rigorous regulation and integrative multi-omics research.

## Figures and Tables

**Table 1:** Childhood Microbiome and Disease Causality.

Study/Taxa	Disease/ Outcome	Type of Evidence	Population	Key Findings	References
TEDDY Study (The Environmental Determinants of Diabetes in the Young)	Type 1 Diabetes (T1DM)	Longitudinal; microbiome sampling from birth in genetically at-risk children	8676 children (United States, European Union)	Microbiome changes (e.g., reduction in *Bifidobacteria*) precede autoantibody appearance	[13]
DIABIMMUNEStudy	Autoimmunity, allergies	Prospective birth cohort; detailed microbiome profiling in high vs. low disease incidence regions	Finland, Estonia, Russia	Early microbial diversity and specific strains (e.g., *Bacteroides dorei*) associated with T1DM risk	[14,15]
*Bacteroides fragilis* and *Bifidobacterium* spp.	Autism Spectrum Disorder (ASD), depression	Experimental: Fecal Microbiota Transplantation (FMT) and probiotic administration from ASD children into mice	ASD vs. neurotypical children	FMT from ASD children induces behavioral changes in mice; probiotic strains reverse some effects	[16,17]
COPSAC (Copenhagen Prospective Study on Asthma in Childhood)	Asthma, allergies	Longitudinal cohort; microbiome profiling and early-life exposures	700+ infants	Early *Streptococcus* colonization linked to wheezing and asthma risk; diversity protective	[18]
*Ruminococcus gnavus*, *Escherichia coli*	Crohn’s disease (early onset)	Longitudinal microbiome tracking and flare-up correlation	Pediatric Crohn’s cohorts	*R. gnavus* bloom precedes flare-ups; *E. coli* expansion seen during inflammation	[19,20]
Gut bacteria	Malnutrition, stunting	Gnotobiotic mouse models colonized with microbiota from malnourished vs. healthy infants	Malnourished Bangladeshi children	Transfer of dysbiotic microbiota causes growth impairment in mice; supplementation with defined strains rescues growth	[21]

**Table 2:** Benefits and drawbacks of several studies on microbiome and disease causation.

Study	Benefits	Drawbacks	References
**TEDDY** (The Environmental Determinants of Diabetes in the Young)	- Large international cohort (United States, Finland, Germany, and Sweden) - Longitudinal design enables tracking from infancy - Comprehensive data collection: biological samples, diet, infections, psychosocial factors - Strong focus on gene–environment interactions in type 1 diabetes mellitus	- Focuses only on genetically at-risk children, limiting generalizability - Expensive and resource-intensive: difficult to replicate - Long follow-up time before appearance of disease outcomes	[13]
**DIABIMMUNE** (Hygiene Hypothesis and Autoimmunity Study)	- Natural experimental setup across differing hygiene environments (Finland, Estonia, Russia) - Emphasizes early microbial exposure and autoimmune risk - Rich microbial sampling: deep metagenomic analysis of the gut microbiome	- Limited to Northern/Eastern Europe, affecting generalizability - Smaller sample size than TEDDY - Shorter follow-up in some sub-cohorts: may limit conclusions on long-term disease outcomes	[14,15]
**COPSAC** (Copenhagen Prospective Studies on Asthma in Childhood)	- Detailed, well-characterized cohorts - Focus on early-life exposures and their link to asthma/allergies - Includes interventional studies (e.g., probiotics) - Extensive biobanking for future OMICS research	- Primarily focused on asthma and allergies - Based in Denmark, limiting diversity of exposures - Also, resource-intensive and complex to replicate	[18]

**Table 3:** Dietary Interventions Based on TEDDY Findings.

Intervention	TEDDY Insight	Impact
Exclusive breastfeeding ≥ 6 months	Enhances microbial diversity, delays autoimmunity	Risk of islet autoimmunity
Introduce solids at 4–6 months	Supports balanced microbiome maturation	Risk of T1DM
Avoid early antibiotics	Preserves protective gut bacteria	Autoantibody risk
High-fiber, SCFA-promoting foods	Enhances immune-regulatory metabolites	T1DM marker development

Legend: SCFA: short-chain fatty acids. T1DM: type 1 diabetes mellitus.

## Data Availability

No new data were created or analyzed in this study.
